# The application of rumen simulation technique (RUSITEC) for studying dynamics of the bacterial community and metabolome in rumen fluid and the effects of a challenge with *Clostridium perfringens*

**DOI:** 10.1371/journal.pone.0192256

**Published:** 2018-02-07

**Authors:** Stefanie U. Wetzels, Melanie Eger, Marion Burmester, Lothar Kreienbrock, Amir Abdulmawjood, Beate Pinior, Martin Wagner, Gerhard Breves, Evelyne Mann

**Affiliations:** 1 Institute for Milk Hygiene, Milk Technology and Food Science, Department for Farm Animals and Veterinary Public Health, University of Veterinary Medicine Vienna, Vienna, Austria; 2 Institute for Physiology, University of Veterinary Medicine Hannover, Hannover, Germany; 3 Institute for Biometry, Epidemiology and Information Processing, University of Veterinary Medicine Hannover, Hannover, Germany; 4 Institute for Food Quality and Food Safety, Research Center for Emerging Infections and Zoonoses (RIZ), University of Veterinary Medicine Hannover, Hannover, Germany; 5 Institute for Veterinary Public Health, Department for Farm Animals and Veterinary Public Health, University of Veterinary Medicine Vienna, Vienna, Austria; Institut Pasteur, FRANCE

## Abstract

The rumen simulation technique (RUSITEC) is a well-established semicontinuous *in vitro* model for investigating ruminal fermentation; however, information on the stability of the ruminal bacterial microbiota and metabolome in the RUSITEC system is rarely available. The availability of high resolution methods, such as high-throughput sequencing and metabolomics improve our knowledge about the rumen microbial ecosystem and its fermentation processes. Thus, we used Illumina MiSeq 16S rRNA amplicon sequencing and a combination of direct injection mass spectrometry with a reverse-phase LC-MS/MS to evaluate the dynamics of the bacterial community and the concentration of several metabolites in a RUSITEC experiment as a function of time and in response to a challenge with a pathogenic *Clostridium perfringens* (*C*. *perfringens*) strain. After four days of equilibration, samples were collected on days 5, 6, 7, 10, 12 and 15 of the steady-state and experimental period. From a total of six fermenters, three non-infected fermenters were used for investigating time-dependent alterations; three fermenters were incubated with *C*. *perfringens* and compared with the non-infected vessels at days 10, 12 and 15. Along the time-line, there was no statistically significant change of the overall bacterial community, however, some phylotypes were enriched at certain time points. A decrease in *Fibrobacter* and *Elusimicrobia* over time was followed by an increase in *Firmicutes* and *Actinobacteria*. In contrast, classical fermentation measurements such as pH, redox potential, NH_3_-N, short chain fatty acids and the concentrations of metabolites determined by metabolomics (biogenic amines, hexoses and amino acids) remained stable throughout the experiment. In response to *C*. *perfringens* addition the concentrations of several amino acids increased. Although the overall bacterial community was not altered here either, some minor changes such as an enrichment of *Synergistetes* and *Bacteroidetes* were detectable over time. In conclusion, both, the bacterial community composition and the metabolome in the RUSITEC system were relatively stable during the experiment.

## Introduction

Detailed knowledge of the ruminal dynamic, anaerobic ecosystem and rumen fermentation processes are the prerequisite to understand basic rumen physiology and nutrition as well as gastrointestinal diseases such as rumen acidosis. Studying factors, which contribute to a modulation *in vivo* underlie timely, environmental fluctuations and host-dependent physiology [1]. The rumen simulation technique (RUSITEC) is a well-established *in vitro* method to simulate and to investigate rumen microbial processes, avoiding animal’s variability in a standardized environment [2]. This method is widely used for studying effects of different diets or feed additives on microbial fermentation patterns, protein synthesis and microbial growth [3, 4]. Although being a highly standardized method (e.g. in temperature, pH and buffer flow) the system is known to differ from *in vivo* conditions regarding absorptive processes, differences in the ratio between liquid and solid materials, lower short chain fatty acid (SCFA) concentrations and protozoal shifts compared to the donor animal [5–7]. It was proposed that the bacterial diversity decreases in the RUSITEC and that the disappearance of ciliates can be traced back to a loss of balance in the bacterial populations [7]. Using real-time PCR, Lengowski and colleagues [8] demonstrated that most changes during the adaptation of the ruminal microbial community to the RUSITEC system occur within in the first 48 h after inoculation, however, may continue for some species. The availability of high-throughput sequencing methods offers the opportunity to investigate alterations in the microbial community and microbial biochemical processes in detail. Belanche and colleagues were the first, who used next generation sequencing methods to evaluate the impact of dietary supplementation in the RUSITEC system [9, 10]. Recently, Duarte and colleagues [11] reported an effect of the sampling day on the liquid-associated microbiota in the RUSITEC using Illumina sequencing.

The rumen microbiota strongly influences the ruminant’s metabolism by the pattern of SCFA and protein formation. While SCFA and NH_3_-N are assessed routinely in most studies [3, 12], metabolomic techniques for the comprehensive analysis of further metabolites, e.g. amino acids and biogenic amines, have only been established a few years ago for rumen fluid [13]. Subsequent studies indicate that the levels of these metabolites are also linked to the feeding regimen and might be related to pathogenic conditions in the rumen [14, 15]. To our knowledge these techniques have not been used in the RUSITEC until now.

Beside of autochthonous bacterial community members, allochthonous microbes constantly pass the rumen deriving from food, water or the environment, of which some can be pathogenic species. Such pathogenic bacteria can cause alterations in the gut microbiome [16, 17]. *Clostridium perfringens (C*. *perfringens)* causes hemorrhagic enteritis in neonatal ruminants, enterotoxemia, jejunal hemorrhage syndrome, abomasal ulcers and tympany or gas gangrene [18, 19]. In adult cattle, *C*. *perfringens* is frequently present in rumen samples [20]. In broiler chicken, *C*. *perfringens* has been reported to alter the intestinal microbiota [21], indicating that it might also be a candidate for modulating the rumen microbiome.

The aim of the study is twofold: First, to provide a detailed investigation on the dynamics in the bacterial microbiota and fermentation products during a 15-day RUSITEC experiment (four days equilibrium, three days steady-state and eight days experimental period) by combining high-throughput sequencing with a targeted quantitative metabolomics approach.

Second, to examine whether the bacterial community and the metabolic profile in the RUSITEC fermenters remain stable during a challenge with *C*. *perfringens* as a model pathogen.

## Materials and methods

### Ethics statement

All procedures involving animals were carried out in accordance with the German legislation on animal welfare. The fistulation of donor cows was approved by the Lower Saxony State Office for Consumer Protection and Food Safety (approval no. 33.42502-05-07A480).

### RUSITEC experiment

The RUSITEC experiment was carried out using six fermenters. The triplicate ‘A-C fermenter’ (samples S1A-S6C) was analyzed in respect to determine time-dependent changes of the microbiota and the metabolome, the triplicate ‘D-F fermenter’ (samples S1D-S6F) was used for the challenge with *C*. *perfringens* as a model pathogen. For all fermenters, rumen content was collected from two non-lactating ruminal fistulated German Holstein cattle (5 years old, body weight approx. 850 kg) owned by the Institute for Physiology. The donor animals were housed on straw bedding and fed hay (7.5 kg/d), a commercial concentrate (500 g/d, Deuka Schaffutter, Deutsche Tiernahrung Cremer, Düsseldorf) and a mineral supplement (75 g/d, VitaMiral Trockensteher, VitaVis GmbH, Münster, Germany). Rumen contents were collected 3 h after morning feeding and separated into liquid and solid content by gauze filtration. At the start of the experiment all fermenters were inoculated with two nylon bags (pore size: 150 μm, Gesellschaft für Analysetechnik HLS, Salzwedel, Germany) in the inner vessel, one containing 70 g of solid rumen content mixed from both animals, one containing the experimental substrate of 5 g hay (2 cm length) and 5 g concentrate, and approximately 750 ml of mixed rumen fluid. After 24 h the rumen content bag was replaced by a substrate bag. Subsequently, bags were changed alternately as described previously [2]. The fermenters were kept in a water bath at 39°C and the inner vessel was slowly moved up and down by an electric motor (six times per minute). Buffer solution (S1 Table) was infused continuously to achieve a liquid turnover of once per day and effluents were collected in conical glass flasks kept on ice. Daily effluent volumes were recorded (data not shown). Fermentation gas was collected in gas bags (Plastigas, Linde AG, Munich Germany) to achieve an air-tight system and after daily change of the substrate bags and sample collection glass flasks were flushed with nitrogen to maintain anaerobic conditions.

### Experimental time schedule and RUSITEC effluent sampling

**Fig 1:**
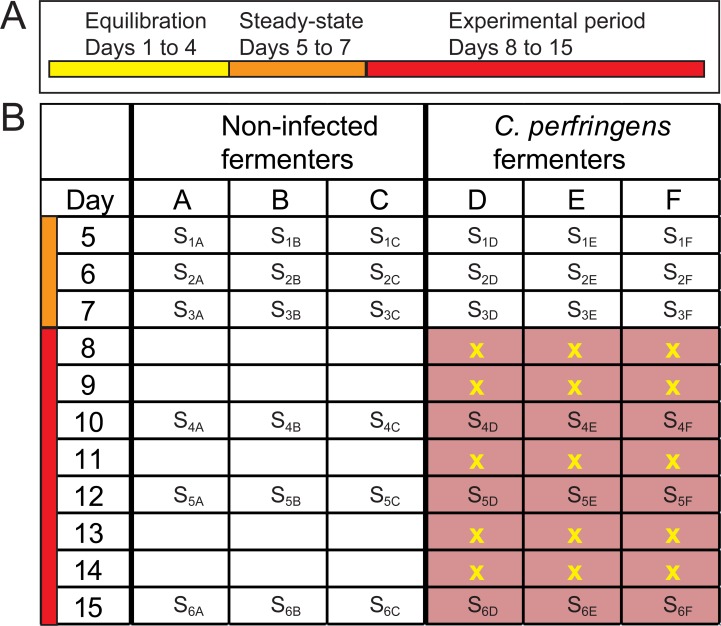
Experimental setup. (A) 4 days equilibration period (yellow), 3 days steady-state period (orange) and 8 days experimental period (red). (B) Three non-infected fermenters from the steady-state- and experimental period are depicted in A-C and three *C*. *perfringens* infected fermenters in D-F. S = RUSITEC effluent sampling, yellow “x” = *C*. *perfringens* inoculation. The red boxes indicate *C*. *perfringens*-spiked fermenters.

The experiment consisted of an equilibration period of four days, a three days steady-state period and an experimental period of eight days (Fig 1A). Throughout the whole experiment pH and redox potential were evaluated daily (digital pH-meter 646, Knick, Berlin, Germany; electrodes: InLab Routine and InLab Redox Pro ORP, Mettler Toledo, Gießen, Germany) to ensure adequate environmental conditions for microbial survival. During the experimental period, 10 ml *C*. *perfringens* inoculum were added to vessels D-F 1 h after feeding at experimental days 8, 9, 11, 13 and 14 at a concentration of 10^8^ CFU/10 ml (Fig 1B). Inoculum was derived from fresh culture containing vegetative cells. Different application intervals were applied to test whether *C*. *perfringens* is able to colonize the fermenters and whether repeated application results in an accumulation of *C*. *perfringens* in the fermenters. Effluent samples (60 ml) were collected during the steady-state period (days 5, 6, 7) and in the experimental period at days 10, 12 and 15 for the assessment of SCFA production and ammonia concentrations as well as for bacterial community and metabolome analysis. Effluent samples were collected before introducing a new feed bag. Samples were frozen immediately at −20°C to minimize metabolite degradation.

### *C*. *perfringens* isolate

*C*. *perfringens* type strain CCUG 1795 T (NCTC 8237/ ATCC 13124) was used as inoculum for the RUSITEC challenge experiment as a model pathogen. The strain was isolated from bovine origin and is positive for the *cpa* toxin gene.

The bacterial suspension was prepared from pure culture of the type strain in thioglycolate broth. The colony forming units (CFU) were estimated using serial dilution of the *C*. *perfringens* culture in sterile NaCl solution (0.85%). The colony number was estimated using plate-casting method. 1 ml of the diluted culture was mixed with 10–15 ml melted Sulfit-Cycloserin (SC) Agar, (Thermo Scientific, Wesel, Germany) at 43–47°C. Then a thin layer of SC Agar was applied. The plates were incubated anaerobically for 20 h ± 2 h at 37°C. The spiking suspension (10 ml) was composed of 10^7^ CFU *C*. *perfringens* per ml.

### DNA extraction, Illumina amplicon sequencing and read processing

The RUSITEC effluent samples (36 samples) and native rumen fluid samples of the donor animals (one of each of the two cows and a 50:50% mix of both) were thawed on ice, vortexed and genomic DNA was extracted from 0.25 ml RUSITEC effluent in duplicate after shaking the tube rigorously. The PowerSoil DNA Isolation Kit (MO BIO Laboratories, Inc., California, USA) was used according to the manufacturer’s protocol (http://www.mobio.com) with following modifications: Mechanical lysis was done for 15 min on a MO BIO Vortex Adapter after 10 min incubation at 70°C. After extraction the duplicate samples were pooled. DNA concentration was determined using the Qubit 2.0 Fluorimeter (Qubit dsDNA BR Assay Kit, Thermo Fisher Scientific, Vienna, Austria). A negative extraction control of the DNA extraction kit was isolated and processed like RUSITEC samples. For sequencing of 40 samples (36 RUSITEC samples, three native rumen fluid samples and one negative extraction control), the V3/4/5 hypervariable region of bacterial 16S rRNA genes was targeted using the primer set 341F (5’-CCTACGGGRSGCAGCAG-3′) [22] and 909R (5′-TTTCAGYCTTGCGRCCGTAC-3′) [23]. Library preparation, Nextera two-step PCR amplification, equimolar pooling of samples and sequencing with a 300bp paired-end reads protocol using an Illumina MiSeq sequencing platform were performed by Microsynth (Balgach, Switzerland). Sequence data were analyzed with the software package QIIME v1.9.1 [24]. A total of 1,367,579 demultiplexed reads was produced and quality controlled. Chimeric sequences were checked with USEARCH 6.1 [25], by comparing sequences against the reference Gold database (http://drive5.com/uchime/gold.fa). Microbial taxonomy was assigned by clustering reads with UCLUST (V.1.2.22q) using Greengenes (V.13.8) as reference database (based on a 97% similarity treshold). OTUs with < 10 sequences were removed, resulting in a definite read count of 997,650 for all downstream analyses. For even sampling in beta diversity calculations, the depth of coverage was set to the lowest number of reads observed in a RUSITEC sample (14,464 reads).

### Direct flow injection, LC-MS/MS compound identification and quantification, short-chain fatty acids (SCFA) and ammonia concentration measurements

Concentrations of amino acids, sugars, acylcarnitines, sphingolipids, glycerophospholipids and biogenic amines were determined using a targeted quantitative metabolomics approach. For this a combination of direct injection mass spectrometry with a reverse-phase LC-MS/MS was applied using the Absolute*IDQ* p150 Kit from BIOCRATES Life Sciences AG (Austria) as described by Saleem and colleagues [26]. One milliliter of all RUSITEC effluent samples was sent to BIOCRATES Life Sciences AG (Austria) for metabolomics analysis. Daily production of SCFA was assessed by multiplying the effluent volume with SCFA concentrations determined by gas chromatography as described previously [27]. Ammonia concentrations were measured photometrically as described by Riede and colleagues [28].

### qPCR of all bacterial counts and artificially added model pathogen *C*. *perfringens*

The standard for qPCRs was prepared with pooled DNA from 12 DNA samples (two samples per time point) with serial dilutions of the purified qPCR products as previously described by Li and colleagues [29]. Standard curves (range 1e+1 – 1e+6 gene copy numbers) were included in each qPCR assay. All amplification reactions and negative controls were pipetted in duplicates. qPCR reactions contained 10 μl 2 × Brilliant III Ultra-Fast SYBR Green qPCR Master Mix (Agilent, Vienna, Austria), 2 μl of each primer (2.5 μM initial concentration), 5 μl of nuclease-free water and 1 μl DNA template. After the reaction, a melting curve with a range of 70 to 95°C with fluorescence measurements at 1°C intervals was done. Total gene copy numbers of 16S rRNA genes were determined using the forward primer 5’-CCTACGGGAGGCAGCAG-3’ (341F) and the reverse primer 5’-ATTACCGCGGCTGCTGG-3’ (518R) [30] (Microsynth, Balgach, Switzerland) with a final concentration of 0.25 μM and 1 μl DNA. qPCR included an initial denaturation step at 95°C for 3 min, followed by 40 cycles of 95°C for 5 s and 61°C for 20 s with a fluorescence measurement at the last step of each cycle. *C*. *perfringens* was quantified with a *plc*-specific qPCR assay [31] using an annealing temperature of 60°C and qPCR conditions as described in Nagpal, et al. [31]. All qPCRs were performed with a Stratagene Mx3000P real-time PCR system (Agilent Technologies, Santa Clara, USA) and results were analyzed using the associated software (Stratagene MxPro, QPCR Software, version 2.00). Information regarding qPCR as recommended by the MIQE guidelines [32] are shown in S2 Table.

### Statistical analysis

To describe differences regarding to sampling time points among diversity indices (Shannon index, Chao 1 estimator) and qPCR results, a linear regression model was applied by using the open access software R [33]. First, the six sampling time points (days 5, 6, 7, 10, 12, and 15, fermenters A-C) were compared among each other. In a second step, fermenters with artificially added *C*. *perfringens* (days 10, 12, and 15, fermenters D-F) were tested against non-infected fermenters (days 10, 12, and 15, fermenters A-C) to verify whether *C*. *perfringens* influences any of these residuals. Data were assessed visually with regards to normal distribution of residuals (histograms and quantile plots). A contrast coefficient among the six different time points (fermenters A-C) and between the *C*. *perfringens* and non-infected group (days 10, 12, and 15) were calculated for each diversity index and qPCR results by using a multiple comparison of means (Tukey contrasts). The contrast calculation was implemented in R using the package multcomp with a significance level attained at *p* ≤ 0.05 (https://cran.r-project.org/web/packages/multcomp/multcomp.pdf).

To test if the bacterial communities differ between the six sampling time points and between the *C*. *perfringens* and non-infected groups, weighted and unweighted UniFrac distances were calculated and analyzed with the compare_categories.py script in QIIME using ANOSIM.

To define bacterial phylotypes and metabolites that were most likely to explain differences between time points and between the *C*. *perfringens* and non-infected group, the metagenomic biomarker discovery tool LEfSe was applied [34] using the Kruskal-Wallis sum-rank test to describe different abundances between groups [34]. LEfSe analysis was performed with a threshold of 2.0 on the logarithmic LDA score for discriminative features with an alpha value < 0.05 for the factorial Kruskal-Wallis test among classes and an all-against-all multi-class analysis strategy.

### Accession number

Illumina MiSeq sequencing data are available in BioProject SRA database under the accession number PRJEB15167.

## Results

### Overall bacterial community composition in the RUSITEC

A total of 1,367,579 demultiplexed reads was produced with the 300 bp paired-end reads protocol using Illumina MiSeq sequencing. After a stringent quality control, a definite read count of 997,650 (73%) remained for all downstream analysis. Considering all 36 RUSITEC samples, 17 bacterial phyla were identified with *Bacteroidetes* and *Firmicutes* being most abundant (91.9% of all reads). In total, 4,071 OTUs were built based on a 0.03 distance level and used for all further downstream analyses. The 50 most abundant OTUs were classified against type strains of the Greengenes database and were listed in Table 1. These type strains were previously described to belong to the commensal rumen microbiota, plants, or environmental samples. The two most abundant OTUs (7.5% and 5.9% relative abundance) were classified as *Prevotella bryantii* and *Prevotella ruminicola* with 100% sequence similarity compared to the best Greengenes type strain hit.

**Table 1:** The 50 most abundant OTUs are shown with the respective relative abundance in the RUSITEC samples, sequence similarity to Greengenes hits, match length and accession number.

OTU	Rel. ab.^1^ (%)	Sim.^2^ (%)	Match length (bp)	Accession No.	Greengenes best hit
2456812	7.50	100	543	NR_028866.1	*Prevotella bryantii* str. B14; DSM 11371^3^
768947	5.92	100	543	AY699286.1	*Prevotella ruminicola* L16^4^
266210	2.19	100	552	EU728750.1	*Megasphaera elsdenii* str. DJF_RP06
312373	2.02	97.61	543	AB501154.1	*Prevotella ruminicola* str. BP1-41^3^
578085	1.77	97.24	543	AB501162.1	*Prevotella ruminicola* str. BP1-80^3^
811229	1.76	98.53	543	AB501173.1	*Prevotella ruminicola* str. AC5-13^3^
84193	1.67	82.14	543	AB255367.1	*Galbibacter mesophilus* str. Mok-17
579278	1.39	91.87	529	NZ_AAXG02000037.1	*Bacteroides capillosus* str. ATCC 29799
576416	1.31	83.06	543	AY643076.1	*Capnocytophaga cynodegmi* str. CIP 103937
4358602	1.17	100	552	AB186315.1	*Lactobacillus mucosae* str. DLS 1003
209705	1.09	82.5	543	AB541983.1	*Prolixibacter bellariivorans* str. JCM 13498
813220	1.09	95.11	552	X81137.1	*Succiniclasticum ruminis* str. SE10
254969	1.04	83.79	543	AB554231.1	*Alistipes onderdonkii* str. JCM 16771
266445	1.00	100	552	EF120372.1	*Lactobacillus amylovorus* str. LAB16
582088	0.86	97.42	543	AB501163.1	*Prevotella ruminicola* str. BP1-90^3^
581091	0.80	99.82	551	Y09434.1	*Schwartzia succinivorans* str. DSM 10502T^3^
137580	0.78	98.36	550	EF120372.1	*Lactobacillus amylovorus* str. LAB16
538223	0.77	99.82	552	EF120372.1	*Lactobacillus amylovorus* str. LAB16
4438135	0.72	82.14	543	AY918928.1	*Prolixibacter bellariavorans* str. F2
343181	0.66	82.99	541	AB078046.1	*Flexibacter canadensis* str. IFO 15130
New.Ref_OTU144	0.65	98.01	552	AB186315.1	*Lactobacillus mucosae* str. DLS 1003
559781	0.63	83.61	543	X97245.1	*Capnocytophaga cynodegmi* str. ATCC 49044
848968	0.59	92.08	543	AB501157.1	*Prevotella ruminicola* str. BP1-60^3^
213576	0.59	96.13	543	AB501162.1	*Prevotella ruminicola* str. BP1-80^3^
831922	0.58	86.2	529	U88891.1	*Desulfotomaculum halophilum* str. SEBR 3139
324495	0.58	88.56	542	AB331896.1	*Paraprevotella clara* str. YIT 11840
New.Ref.OTU379	0.58	98.19	552	EU163503.1	*Streptococcus lutetiensis* str. 907
New.Ref.OTU373	0.57	98.73	552	AB186315.1	*Lactobacillus mucosae* str. DLS 1003
1082539	0.57	100	552	EU163503.1	*Streptococcus lutetiensis* str. 907
738975	0.56	87.07	549	DQ833400.1	*Sphaerochaeta* sp. str. TQ1
272580	0.55	93.74	543	AB501163.1	*Prevotella ruminicola* str. BP1-90^3^
592852	0.55	96.32	543	AB501173.1	*Prevotella ruminicola* str. AC5-13^3^
637375	0.54	89.25	549	DQ833400.1	*Sphaerochaeta* sp. str. TQ1
635310	0.50	83.61	543	NR_025910.1	*Rikenella microfusus* str. Q-1; ATCC 29728
New.Ref.OTU86	0.49	82.32	543	AF118419.1	*Coenonia anatina* str. 726–82
New.Ref.OTU266	0.48	98.19	552	AB186315.1	*Lactobacillus mucosae* str. DLS 1003
New.Ref.OTU56	0.47	82.66	548	EU281854.1	*Eubacterium* sp. str. F1^3^
628517	0.45	86.09	532	DQ903989.1	*Cytophaga* sp. PRPR22
330492	0.43	91.08	527	X76161.1	*Clostridium aminobutyricum* str. DSM 2634
338757	0.42	98.91	552	AB186315.1	*Lactobacillus mucosae* str. DLS 1003
589852	0.42	100	526	NR_026315.1	*Pseudobutyrivibrio ruminis* str. DSM 9787^3^
546360	0.40	95.76	543	AB501173.1	*Prevotella ruminicola* str. AC5-13^3^
237728	0.40	82.5	543	GU470889.1	*Capnocytophaga* sp. oral taxon 329 str. F0087
144161	0.39	94.84	543	AF218619.1	*Prevotella ruminicola* str. TC2-28^3^
348764	0.39	86.74	528	AB186360.1	*Clostridium* sp. str. EBR-02E-0046
New.Ref.OTU303	0.39	84.12	548	AF183405.1	*Prevotella tannerae* str. 89-9-1
New.Ref.OTU159	0.39	85.45	543	AB510702.1	*Bacteroides helcogenes* str. JCM 6297
732053	0.38	84.67	548	EU281854.1	*Eubacterium* sp. str. F1^3^
548324	0.37	84.31	548	EU281854.1	*Eubacterium* sp. str. F1^3^
262849	0.37	96.01	552	NR_026450.1	*Ruminobacter amylophilus* str. H 18; DSM 1361
353910	0.36	86.53	527	AJ229251.1	*Clostridium* sp. str. FCB90-3
New.Ref.OTU97	0.36	85.5	545	AB547676.1	*Prevotella buccalis* str. JCM 12246
99436	0.35	99.64	552	AB198428.1	*Selenomonas ruminantium* str. S8^3^
New.Ref.OTU512	0.35	98.55	552	EU163503.1	*Streptococcus lutetiensis* str. 907

^1^ Rel. ab. = Relative abundance

^2^ Sim. = Sequence similarity to best Greengenes hit

^3^ Best Greengenes hit isolated from the rumen.

^4^ Best Greengenes hit isolated from the gastro-intestinal tract of ruminants.

Additionally, we sequenced three native rumen fluid samples. *Firmicutes* and *Bacteroidetes* were the most abundant phyla (56.4% and 38.5%, respectively). In total, 91% of the OTUs detected in the RUSITEC samples were also detected in the native rumen fluid samples including all OTUs with a relative abundance of > 0.8%. A total of 70.5% of the OTUs were overlapping between the RUSITEC samples and the native rumen fluid samples. Overall bacterial community composition of the native rumen fluid samples differed statistically significantly from the RUSITEC samples (ANOSIM; *p* = 0.001).

#### Bacterial community composition at different time points did not change significantly in the RUSITEC

**Fig 2:**
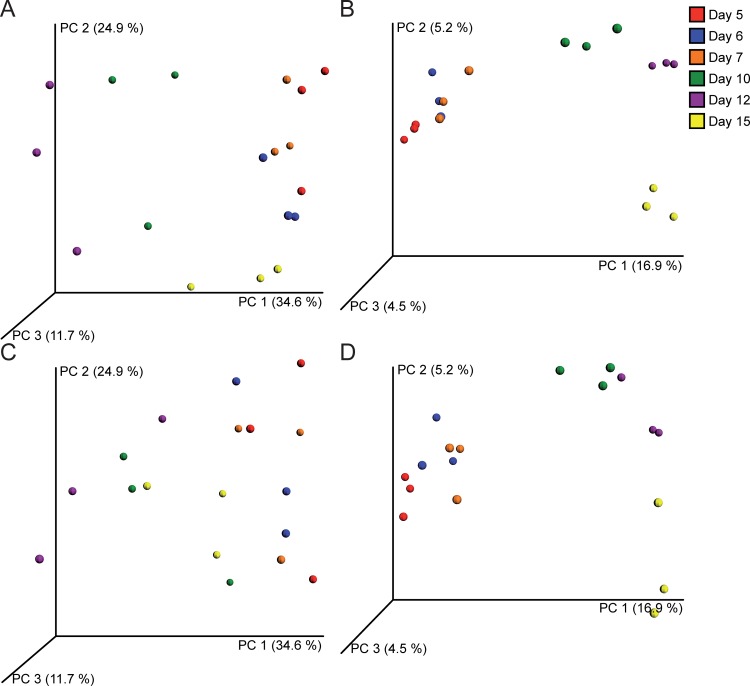
UniFrac distances of amplicon sequencing data depicted as PCoA Plot with sampling days shown in different colors. The percent variation explained by each principal coordinate is indicated by the axes. A: weighted and B: unweighted UniFrac with only non-infected fermenters (Fig 1B, fermenters A-C). C: weighted and D: unweighted UniFrac with fermenters used for the *C*. *perfringens* spiking experiment (Fig 1B, fermenters D-F). Only fermenters at days 10, 12, and 15 are spiked with *C*. *perfringens*. Native rumen fluid samples of donor animals are included in each panel (Native rumen fluid).

The bacterial communities within the triplicates (fermenters A-C or fermenters D-F, tested for each time point) did not differ among each other (ANOSIM; *p* = 0.518). The number of OTUs decreased slightly at the end of the experiment (day 5 compared with day 12 and day 15, *p* = 0.044 and *p* = 0.027, respectively), however, there was no statistically significant difference if compared along the time line. Furthermore, the Shannon index did not differ among time points. There was a statistically significant increase of estimated species richness (Chao 1 estimator) from day 5 to day 6 (*p* = 0.033) and a statistically significant decrease from day 7 to day 10 (*p* = 0.048) (Table 2). Bacterial community compositions at different time points did not change according to the weighted UniFrac distances (ANOSIM for day 5 compared to day 6, day 6 compared to day 7, day 7 compared to day 10, day 10 compared to day 12, and day 12 compared to day 15, *p* = 0.125, *p* = 0.105, *p* = 0.115, *p* = 0.094, and *p* = 0.104, respectively, Fig 2A). However, a continuous community shift was observed in the unweighted UniFrac analysis at sampling days 7 to 15 (Fig 2B), indicating a stable bacterial community composition from days 5 to 7 (steady-state period) and a shift between the latter three days compared to days 10, 12 and 15 as well as within days 10, 12 and 15. The native rumen fluid samples clustered separately from the RUSITEC samples.

**Table 2:** Statistical analysis of OTU richness and diversity of non-infected fermenters A-C with regard to sampling day.

	Sampling day						
	Steady-state period			Experimental period			
	5	6	7	10	12	15	*p*-value
observed OTUs	1673 ± 102^ac^	1690 ± 44^a^	1704 ± 116^ac^	1523 ± 75^bc^	1486 ± 46^b^	1390 ± 103^b^	< 0.001*
Chao 1	2652 ± 61^bc^	2807 ± 58^a^	2735 ± 43^ab^	2543 ± 110^ce^	2362 ± 77^de^	2241 ± 99^d^	< 0.001*
Shannon	8.01 ± 0.27	8.07 ± 0.09	8.08 ± 0.24	8.03 ± 0.29	8.17 ± 0.14	7.77 ± 0.39	NS

*Time contrasts were tested between all groups and non-significant differences were marked with same letters.

NS = not statistically significant.

**Fig 3:**
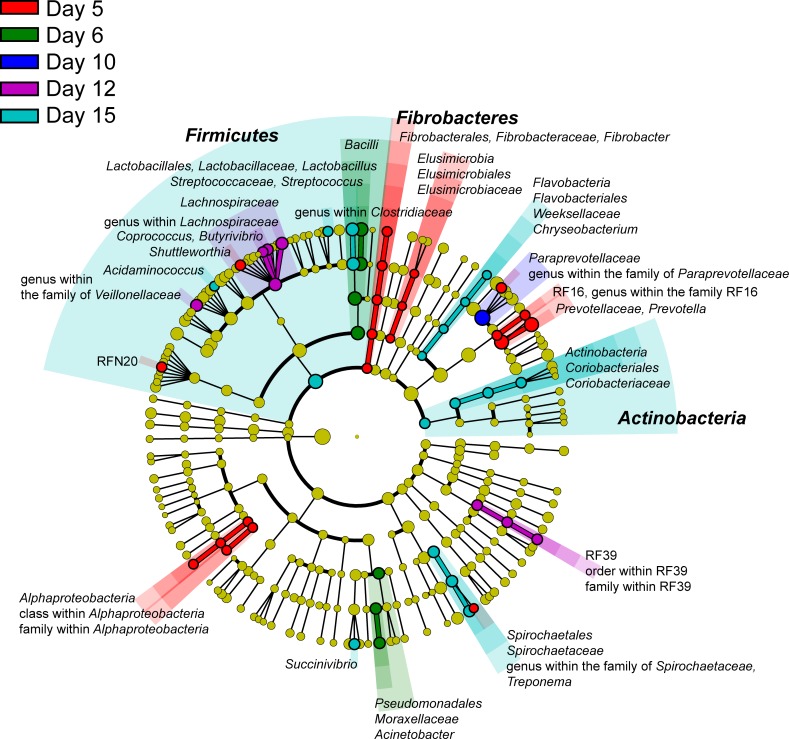
LEfSe analysis. Phylotypes which were statistically significantly enriched at a certain time point were highlighted in color in the cladogram. To investigate shifts along the time line only the non-infected fermenters A-C were used.

To investigate shifts along the time line only the non-infected fermenters A-C were used. In total, 54 phylotypes were found to be statistically significantly enriched along the time line at a certain time point (Fig 3). At day 5, e.g. the phylum *Fibrobacteres*, the classes of *Alphaproteobacteria* and *Elusimicrobia* and the family *Prevotellaceae* were enriched compared to other sampling days. At day 6, the class of *Bacilli* and the order of *Pseudomonadales* were increased and at day 7, the family *Paraprevotellaceae* was enriched compared to other sampling days. At day 12, e.g. *Lachnospiraceae* was increased. At day 15, the highest number of shifts was observed: The phyla *Firmicutes* and *Actinobacteria*, as well as *Flavobacteriales* and *Spirochaetales* were enriched compared to other sampling days.

The metabolic profile, pH and redox potential were summed up in Table 3. From 14 amino acids tested, 11 passed the detection limit and were analyzable. All of them remained stable along the sampling period. From 6 biogenic amines which were tested, only putrescine decreased statistically significantly at day 15 (*p* = 0.048). SCFA, ammonia levels, redox potential and pH also remained stable over the trial.

**Table 3:** Metabolite concentrations, redox potential and pH of non-infected fermenters A-C with regard to sampling time points.

	Sampling day						
	Steady-state period			Experimental period			
	5	6	7	10	12	15	*p*-value
Alanine [μM]	4.55 ± 1.94	7.56 ± 1.96	5.39 ± 0.64	6.13 ± 0.64	7.51 ± 0.88	3.77 ± 0.69	NS
Aspartate [μM]	12.23 ± 1.10	12.33 ± 1.70	12.73 ± 0.31	12.73 ± 0.65	15.17 ± 1.31	12.5 ± 0.56	NS
Citrulline [μM]	1.93 ± 0.15	2.14 ± 0.46	2.15 ± 0.40	2.11 ± 0.20	2.29 ± 0.12	2.20 ± 0.48	NS
Glutamate [μM]	21.4 ± 3.74	21.20 ± 4.23	20.03 ± 1.65	19.40 ± 1.23	22.10 ± 1.81	17.30 ± 1.41	NS
Isoleucine [μM]	0.60 ± 0.07	1.35 ± 0.36	1.34 ± 0.61	1.58 ± 0.02	1.94 ± 0.41	1.03 ± 0.41	NS
Leucine [μM]	1.75 ± NA	NA	NA	3.26 ± NA	NA	NA	NA
Lysine [μM]	1.93 ± 0.78	3.54 ± 2.42	4.73 ± 2.14	4.58 ± 1.35	5.08 ± 1.58	2.32 ± 0.92	NS
Ornithine [μM]	1.14 ± 0.40	0.90 ± 0.01	NA	NA	NA	NA	NS
Phenylalanine [μM]	NA	0.59 ± 0.14	0.49 ± 0.35	0.62 ± 0.27	0.67 ± 0.40	0.22 ± 0.15	NS
Proline [μM]	1.43 ± NA	2.10 ± 0.49	1.88 ± 0.50	1.96 ± 0.22	2.31 ± 0.81	1.76 ± 0.50	NS
Serine [μM]	1.09 ± NA	1.08 ± NA	1.66 ± 0.74	2.03 ± NA	1.81 ± 0.20	1.41 ± 0.55	NS
Threonine [μM]	0.78 ± 0.02	1.12 ± 0.42	0.84 ± 0.34	1.20 ± 0.17	1.31 ± 0.49	0.86 ± 0.12	NS
Tyrosine [μM]	NA	NA	NA	0.53 ± NA	0.52 ± NA	NA	NA
Valine [μM]	NA	NA	NA	NA	0.63 ± NA	NA	NA
Histamine [μM]	0.06 ± 0.05	0.15 ± 0.15	NA	0.06 ± NA	0.03 ± NA	NA	NS
Methionine sulfoxide [μM]	0.56 ± 0.16	0.46 ± 0.11	0.54 ± 0.33	0.61 ± 0.26	0.69 ± 0.29	0.57 ± 0.15	NS
Phenylethylamine [μM]	3.26 ± 0.51	3.40 ± 0.21	3.25 ± 0.07	3.87 ± 0.34	3.27 ± 0.30	3.57 ± 0.49	NS
Putrescine [μM]	2.48 ± 0.44	2.72 ± 0.39	1.89 ± 0.12	2.20 ± 0.38	2.54 ± 0.05	1.86 ± 0.08	0.048
Serotonin [μM]	0.08 ± NA	0.08 ± 0.01	0.08 ± 0.00	0.09 ± 0.01	0.08 ± 0.01	0.08 ± 0.01	NS
Spermidine [μM]	1.84 ± 0.23	1.86 ± 0.07	1.61 ± 0.27	2.06 ± 0.35	1.92 ± 0.24	1.58 ± 0.22	NS
Hexose [μM]	44.00 ± 6.11	48.20 ± 7.88	45.00 ± 7.23	52.00 ± 13.97	47.83 ± 7.64	39.55 ± 3.04	NS
pH	6.84 ± 0.04	6.82 ± 0.01	6.8 ± 0.05	6.74 ± 0.06	6.76 ± 0.04	6.83 ± 0.04	NS
Redox potential [mV]	-271 ± 14	-275 ± 8	-264 ± 7	-294 ± 15	-265 ± 11	-259 ± 6	NS
Ammonia [mM]	7.98 ± 0.45	7.14 ± 0.20	7.12 ± 0.23	7.74 ± 0.62	8.19 ± 0.14	7.50 ± 0.17	NS
C2 [mmol/d]	16.70 ± 1.29	15.66 ± 1.41	15.57 ± 1.06	18.50 ± 1.33	16.58 ± 2.24	14.51 ± 1.85	NS
C3 [mmol/d]	7.01 ± 0.80	6.57 ± 0.87	6.17 ± 0.34	7.76 ± 0.77	6.83 ± 0.96	5.70 ± 1.01	NS
C4 [mmol/d]	4.67 ± 0.71	5.29 ± 0.66	5.51 ± 0.92	6.64 ± 0.17	6.26 ± 0.60	5.51 ± 1.01	NS
iC5 [mmol/d]	0.86 ± 0.10	0.90 ± 0.08	0.90 ± 0.09	0.85 ± 0.09	0.77 ± 0.16	0.77 ± 0.10	NS
C5 [mmol/d]	1.39 ± 0.11	1.90 ± 0.19	1.85 ± 0.34	2.57 ± 0.20	2.43 ± 0.24	2.15 ± 0.44	NS

NA = not available, concentration was below the detection limit.

NS = not statistically significant.

Total gene copy numbers, determined by qPCR, differed statistically significantly among sampling time points (Table 4). The increase of gene copy numbers was statistically significant at days 12 and 15 compared with days 5, 6, 7 and 10 (day 12 compared to days 5, 6, 7 and 6: *p* = 0.007, *p* = 0.019, *p* = 0.004, *p* = 0.001, and day 11 compared to days 1, 2, 3 and 6: *p* = 0.024, *p* = 0.038, *p* = 0.019, *p* = 0.022, respectively).

**Table 4:** Total bacteria qPCR results of the control fermenters (fermenter A-C).

	Sampling day						
	Steady-state period			Experimental period			
	5	6	7	10	12	15	*p*-value
Gene copy numbers	5.38E+07^b^	6.04E+07^b^	5.15E+07^b^	5.70E+07^b^	8.50E+07^a^	1.04E+08^a^	< 0.001*
Standard deviation	9.99E+06	1.05E+07	9.06E+06	4.08E+06	3.90E+06	2.22E+07	

*Time contrasts were tested between all groups and non-significant differences were marked with same letters.

### *C*. *perfringens* impacts certain phylotypes but not the overall bacterial community composition and metabolome

The *C*. *perfringens* strain, which was added to fermenters D-F during the experimental period, was detected to be the 113 most abundant OTU with a relative abundance of 0.80%, 0.36% and 0.56% at days 10, 12 and 15 in the sequencing approach. The OTU was not detected in any un-spiked fermenter.

**Fig 4:**
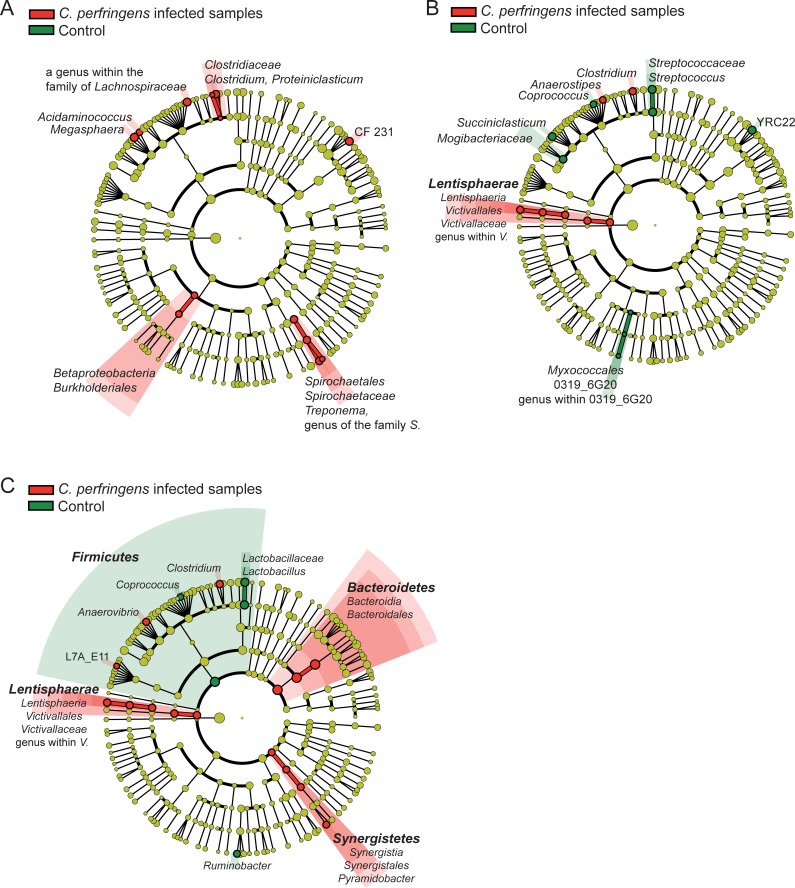
LEfSe analysis. Phylotypes which are statistically significantly enriched in the non-infected vessels or in the *C*. *perfringens* infected vessels were highlighted in color in the cladogram. A = day 10, B = day 12, and C = day 15.

Bacterial community compositions did not differ between non-infected and *C*. *perfringens* fermenters (S4A-S4C versus S4D-S4F, S5A-S5C versus S5D-S5F, S6A-S6C versus S6D-S6F; ANOSIM; *p* = 1.000, *p* = 0.589, and *p* = 0.101, respectively). Moreover, *C*. *perfringens* infected samples did not cluster separately in the weighted and unweighted UniFrac analyses (S1 Fig) and the shifts over time, depicted in Fig 2C and 2D, were similar to non-infected fermenters. OTU richness and diversity did not differ between non-infected and infected fermenters at any sampling time point (S3 Table). In total, merely 13, 16 and 20 phylotypes were found to be differentially enriched between the non-infected and the *C*. *perfringens* fermenters at days 10, 12 and 15, respectively (Fig 4). Enrichments varied between sampling days, except of *Lentisphaerae* which increased in *C*. *perfringens* infected samples at days 12 and 15 and *Coprococcus* which was enriched in non-infected samples at days 12 and 15. On phylum level *Synergistetes* and *Bacteroidetes* were enriched at day 15 in infected fermenters, while *Firmicutes* were more abundant in non-infected fermenters.

Differences of some metabolites between the fermenters before spiking of fermenters (non-infected versus un-spiked infected fermenters: S1A-S1C versus S1D-S1F, S2A-S2C versus S2D-S2F or S3A-S3C versus S3D-S3F) were already statistically significant: acetate, propionate, butyrate, isobutyrate, valerate, ammonia, methionine sulfoxide, phenylethylamine, putrescine, aspartate, glutamate, spermidine, isoleucine, citrulline, lysine, proline, alanine, and hexose. For the statistical comparison of metabolites in non-infected versus infected fermenters, these metabolites were excluded from the analyses.

Statistically significant differences in metabolites in the *C*. *perfringens* fermenters compared to the non-infected fermenters were summarized in Table 5. The concentration of six amino acids (valine, ornithine, leucine, tyrosine, phenylalanine and threonine) increased statistically significantly in the *C*. *perfringens* fermenters compared to the non-infected fermenters.

**Table 5:** Metabolite concentration between non-infected (A-C) and infected fermenters (D-F).

		Fermenters						
		A-C			D-F			*p*-value
Leucine [μM]	Sampling day 10	3.26	±	0.02	6.49	±	2.04	NS
	Sampling day 12	NA	±	NA	7.76	±	0.76	0.037
	Sampling day 15	NA	±	NA	5.33	±	0.88	0.037
Ornithine [μM]	Sampling day 10	NA	±	NA	1.86	±	0.98	NS
	Sampling day 12	NA	±	NA	1.65	±	0.62	0.037
	Sampling day 15	NA	±	NA	1.10	±	0.55	NS
Phenylalanine [μM]	Sampling day 10	0.62	±	0.27	3.44	±	1.38	0.050
	Sampling day 12	0.67	±	0.40	3.93	±	0.45	0.050
	Sampling day 15	0.22	±	0.15	2.48	±	0.77	0.050
Threonine [μM]	Sampling day 10	1.20	±	0.17	2.42	±	0.89	0.050
	Sampling day 12	1.31	±	0.49	2.38	±	0.50	0.050
	Sampling day 15	0.86	±	0.12	1.40	±	0.34	0.050
Tyrosine [μM]	Sampling day 10	0.53	±	NA	1.42	±	0.94	0.046
	Sampling day 12	0.52	±	NA	1.66	±	0.45	0.046
	Sampling day 15	NA	±	NA	0.78	±	0.27	0.037
Valine [μM]	Sampling day 10	NA	±	NA	7.06	±	3.45	0.037
	Sampling day 12	NA	±	NA	7.67	±	1.63	0.046
	Sampling day 15	NA	±	NA	4.14	±	1.29	0.037

NA = not available, concentration was below the detection limit.

NS = not statistically significant.

Consistent with sequencing analysis, the used *C*. *perfringens* strain was detected only in spiked infected fermenters via qPCR at sampling days 10, 12, and 15. Between 1.6 × 10^6^ and 6.1 × 10^6^ gene copy numbers per ml were detected. Gene copy numbers of fermenters did not differ among sampling days 10, 12, and 15 (*p* = 0.579, *p* = 0.839, and *p* = 0.394, for day 10 compared to day 12, day 10 compared to 15, and day 12 compared to day 15, respectively). Total gene copy numbers, determined with qPCR, did not differ either between *C*. *perfringens* fermenters and non-infected fermenters.

## Discussion

Since 1977, the RUSITEC system has been used and well established to evaluate certain rumen conditions *in vitro* [2]. Previous studies using single strand conformation polymorphism and qPCR indicated, that although the protozoa population decreases strongly in this *in vitro* system, the bacterial and the archaeal population are able to adapt to the RUSITEC system [8, 35]. Therefore, several fingerprint methods and qPCR have been intensively used for comparing bacterial communities within the RUSITEC system [28, 36, 37]. Belanche et al. [9, 10] were the first, who used next generation sequencing methods to evaluate the impact of dietary supplementation in the RUSITEC system and Duarte and colleagues [11] detected a shift in the microbiome between day 5 and 10 of a RUSITEC trial by using next generation sequencing. Our study investigated the stability of the bacterial community structure as well as its metabolome in the RUSITEC system along the time line in shorter intervals then in the latter studies. As a further challenge *C*. *perfringens* was added as a model organism to study the effect of a common pathogen on the stability of the bacterial community and metabolome in the RUSITEC system.

*Bacteroidetes* and *Firmicutes* were the predominant phyla detected in the RUSITEC samples, which is in agreement with the studies by Duarte et al. [11, 38], most *in vivo* studies [39–42] and the native rumen fluid samples of the two donor cows. The high overlap of OTUs found in the RUSITEC samples and in the native rumen fluid samples indicates a high degree of stability of rumen fluid bacteria in the RUSITEC system. However, the transfer of the rumen fluid from the rumen to the *in-vitro* system, different diets and the lack of the barn environment might have led to statistically significant differences between the bacterial communities of the native rumen fluid and RUSITEC samples.

Since the RUSITEC is a continuous *in vitro* system, besides the general similarity of the bacterial community, its stability has to be considered. In cows continuously fed the same diet, the bacterial community structure in the rumen remains relatively stable over time [29, 43]. In this study, the fluctuation of 54 phylotypes detected over the time line indicated a slight variation of the bacterial community in the RUSITEC system. On phylum level an initial decrease in the relative abundance of *Fibrobacteres* was observed, while the percentage of *Firmicutes* and *Actinobacteria* increased at the end of the experiment. In the rumen, *Fibrobacter succinogenes* is a specialized cellulose degrader [44], which is reduced under high levels of starch in the diet [42, 45]. Therefore, the higher concentrate to roughage ratio in the RUSITEC substrate bags compared to the donor animal diet might contribute to the decrease in the abundance of *Fibrobacteres*. This hypothesis is supported by the reduced abundance of *Elusimicrobia*, which are able to digest lignocellulose and are also enriched in hay diets [10, 46, 47]. Overall, several *Firmicutes* phylotypes were enriched at differing experimental days. The final increase of the entire phylum *Firmicutes* started with an enrichment of *Lachnospiraceae*, one of the most abundant families within this phylum in rumen samples [42, 48]. The phylum *Firmicutes* contains bacteria identified as cellulose or hemicellulose degraders, e.g. families *Lachnospiraceae* (genus *Butyrivibrio*) and *Ruminococcaceae* [49–51]. In contrast to *Fibrobacter*, *Firmicutes* are also capable of breaking down starch [50] and might to some extent replace the *Fibrobacteres*. *Actinobacteria* are more abundant in cattle subjected to a rumen acidosis challenge [12, 52, 53] and in cattle fed a starch-rich diet [42] and might also benefit from the higher concentrate proportion in the substrate bags. To distinguish whether the observed shifts are linked to the diet change between animal and RUSITEC or directly related to the *in vitro* system further studies are required comparing samples from donor animals and the RUSITEC receiving the same diet. Here, the overall bacterial community structure within the current RUSITEC experiment did not exhibit statistically significant changes along the time line, which is in contrast to the study by Duarte et al. [11]. In our study, the putative loss of lowly abundant phylotypes is reflected by a shift in the unweighted UniFrac analysis, whereas no clear clustering of sampling days could be detected in the weighted UniFrac analysis.

Fermentation parameters such as pH, redox potential and concentrations of NH_3_-N and SCFAs were not altered by the enrichment of individual phylotypes. Among 21 metabolites detected in the metabolome analysis, only putrescine varied statistically significantly along the time line. However, no clear trend was observed as the lowest concentrations were observed at days 7 and 15. The stability of the overall bacterial community together with the stability of the metabolome let conclude that the time-restricted enrichment of phylotypes is rather a stochastic event resulting from the constant interaction between bacteria and metabolites, than a constant community shift.

The ingestion of putative pathogens may result in an enrichment of these organisms in the ruminal bacterial community [16, 54], which might cause major shifts in the community structure. In this study, we observed the establishment of *C*. *perfringens* as a model pathogen in the infected fermentation vessels, which reflects the risk of a putative successful establishment of this pathogen in the rumen under *in vivo* conditions with healthy animals. However, the stable abundance of *C*. *perfringens* (0.80%, 0.36% and 0.56% at days 10, 12 and 15) in this RUSITEC experiment let us hypothesize that the bacterium survives the passage to the rumen in cows, but cannot colonize it.

In infected fermenters, the concentrations of several amino acids were elevated. *C*. *perfringens* produces extracellular proteases and may take up amino acids [55]. Therefore, the increase in amino acid concentrations might have been due to the proteolytic activity of *C*. *perfringens*. The overall bacterial community structure was not significantly altered by addition of *C*. *perfringens* to the fermenters and only few significantly enriched phylotypes were detected by LEfSe analysis. These changes might be rather a result of the changes in the metabolome or variations within the bacterial community of the RUSITEC than a direct interaction with the added *C*. *perfringens*.

Some metabolites could not be evaluated for effects of *C*. *perfringens* addition due differences in the steady-state period. As all differences within metabolites except for methionine sulfoxide were only present on day 5 of the steady-state period, an extension of the equilibration period might help to overcome this issue.

In summary, despite some minor phylotype shifts as a function of time, the overall bacterial community in the RUSITEC fermenters was stable and the addition of a model pathogenic bacterium only affected the metabolome.

## Supporting information

S1 Fig UniFrac distances of amplicon sequencing data depicted as PCoA Plot with sampling days shown in different colors. The percent variation explained by each principal coordinate is indicated by the axes. The fermenters used for the infection with C. perfringens (fermenters D-F) are marked with an asterisk to distinguish them from the non-infected fermenters. Only infected fermenters at days 10, 12, and 15 are spiked with C. perfringens. A: weighted, B: unweighted UniFrac with all fermenters (including “infected” samples). Native rumen fluid samples of donor animals are included in both panels (Native rumen fluid).
(EPS)

S1 Table Chemical composition of the buffer solution. (DOCX)

S2 Table Primers used for qPCR and MIQE guidelines checklist for evaluation of qPCR primers. (DOCX)

S3 Table **OTU richness and diversity between non-infected (A-C) and infected vessels (D-F)**.
(DOCX)
