# Supplementary material for: The application of rumen simulation technique (RUSITEC) for studying dynamics of the bacterial community and metabolome in rumen fluid and the effects of a challenge with Clostridium perfringens
Source: PLoS One. 2018 Feb 7;13(2):e0192256. doi: 10.1371/journal.pone.0192256 (PMC5802913; doi:10.1371/journal.pone.0192256)
Supplement: S1 Table — (DOCX) [file pone.0192256.s002.docx]

S1 Table. Chemical composition of the buffer solution.

| Ingredient | Concentration [mM] |
| --- | --- |
| NaCl | 28.00 |
| KCl | 7.69 |
| HCl (1 N) | 0.50 |
| Ca_2_Cl ∙ 2 H_2_O | 0.22 |
| MgCl_2_ ∙ 6 H_2_O | 0.63 |
| NH_4_Cl | 5.00 |
| Na_2_HPO_4_ ∙ 12 H_2_O | 10.00 |
| NaH_2_PO_4_ ∙ H_2_O | 10.00 |
| NaHCO_3_ | 97.90 |
