# Supplementary material for: The application of rumen simulation technique (RUSITEC) for studying dynamics of the bacterial community and metabolome in rumen fluid and the effects of a challenge with Clostridium perfringens
Source: PLoS One. 2018 Feb 7;13(2):e0192256. doi: 10.1371/journal.pone.0192256 (PMC5802913; doi:10.1371/journal.pone.0192256)
Supplement: S2 Table — (DOCX) [file pone.0192256.s003.docx]

S2 Table. Primers used for qPCR and MIQE guidelines checklist for evaluation of qPCR primers.

| **OTU/ Target group** | **Sequences of primers (5' - 3')** | | **AT^1^** | | | **SYBR Standards RSq** | **SYBR, Y** | **Efficiency [%]** | **Linear dynamic range tested** | **Amplicon size (bp)** | **Reference** |  |
| --- | --- | --- | --- | --- | --- | --- | --- | --- | --- | --- | --- | --- |
|  |  |  |  |  |  |  |  |  |  |  |  |  |
| All bacteria | CCTACGGGAGGCAGCAG | | 61 | | | 0.999 | -3.359*LOG(X) + 32.27 | 98.5 | 4.49E+01 to | 189 | Muyzer et al., 1993 |  |
|  | ATTACCGCGGCTGCTGG | |  | | |  |  |  | 4.49E+07 |  |  |  |
| *plc* gene | CCGTTGATAGCGCAGGACA | | 60 | | | 0.998 | -3.350*LOG(X) + 37.52 | 98.8 | 1.39E+03 to | 219 | Nagpal et al., 2015 |  |
|  | CCAACTATGACTCATGCTAGCA | |  | | |  |  |  | 1.39E+06 |  |  |  |
| ^1^  Annealing temperature in °C. | | | | | | | | | | | |  |
| **Item** | | **Importance** | | **Status** | **Remarks** | | | | | | | |
| **Experimental design** | |  | |  |  | | | | | | | |
| definition of experimental and control groups | | E | | OK | Non-infected (fermenters A-C), Infected (fermenters D-F) | | | | | | | |
| number within each group | | E | | OK | three biological replicates per sample and time point | | | | | | | |
| assay carried out by core lab or investigator's lab? | | D | | OK | Investigator's lab | | | | | | | |
| acknowledgement of authors' contributions | | D | | OK | done | | | | | | | |
| **Sample** | |  | |  |  | | | | | | | |
| description | | E | | OK | DNA isolated from RUSITEC effluent | | | | | | | |
| volume/mass of sample processed | | D | | OK | 2 x 0.25 ml RUSITEC effluent for each sample | | | | | | | |
| microdissection or macrodissection | | E | | - | not relevant | | | | | | | |
| processing procedure | | E | | OK | DNA isolation with PowerSoil DNA isolation kit | | | | | | | |
| if frozen - how and how quickly? | | E | | OK | within 15 minutes frozen at -20°C | | | | | | | |
| if fixed - with what, how quickly? | | E | | OK | not fixed | | | | | | | |
| sample storage conditions and duration | | E | | OK | 3 months stored at -20°C, after thawing all samples were processed within 5 minutes | | | | | | | |
| **Nucleic acid extraction** | |  | |  |  | | | | | | | |
| procedure and/or instrumentation | | E | | OK | PowerSoil DNA isolation kit, with mechanical lysis | | | | | | | |
| name of kit and details of any modifications | | E | | OK | PowerSoil DNA Isolation Kit (MO BIO Laboratories, Inc., California, USA) - mechanical lysis was done for 15 min on a MO BIO Vortex Adapter after 10 min incubation at 70°C | | | | | | | |
| source of additional reagents used | | D | | OK | DNA eluted in DEPC-treated water | | | | | | | |
| details of DNase or RNase treatment | | E | | OK | no treatment | | | | | | | |
| contamination assessment (DNA or RNA) | | E | | OK | negative controls were included in all qPCR assays | | | | | | | |
| nucleic acid quantification | | E | | OK | Qubit 2.0 Fluorimeter (Qubit dsDNA BR Assay Kit, Thermo Fisher Scientific, Vienna, Austria) and NanoDrop (Thermo Fisher Scientific) | | | | | | | |
| instrument and method | | E | | OK | Qubit 2.0 Fluorimeter (Qubit dsDNA BR Assay Kit, Thermo Fisher Scientific, Vienna, Austria) and NanoDrop (Thermo Fisher Scientific) | | | | | | | |
| purity (a260/a280) | | D | | OK | 2.1 (median value for all samples). The DNA concentrations were <10ng/µl. Results are not trustable (see manual under “common problems”: http://www.mgp.cz/files/nanodrop/manualy/pomer_cistoty.pdf). | | | | | | | |
| yield | | D | | OK | 0.67-5.43 ng/µl | | | | | | | |
| RNA integrity method/instrument | | E | | - | not relevant | | | | | | | |
| RIN/RQI or Cq of 3' and 5' transcripts | | E | | - | not relevant | | | | | | | |
| electrophoresis traces | | D | | - | not relevant | | | | | | | |
| inhibition testing (Cq dilutions, spike or other) | | E | | OK | spike, no inhibition was observed | | | | | | | |
| **qPCR target information** | |  | |  |  | | | | | | | |
| if multiplex, efficiency and LOD of each assay. | | E | | OK | no multiplexing | | | | | | | |
| sequence accession number | | E | | - |  | | | | | | | |
| location of amplicon | | D | | OK | see references for all bacteria and plc gene | | | | | | | |
| amplicon length | | E | | OK | see all bacteria and plc gene | | | | | | | |
| in silico specificity screen (blast, etc) | | E | | OK | see references for all bacteria and plc gene | | | | | | | |
| pseudogenes, retropseudogenes or other homologs? | | D | | - | not relevant | | | | | | | |
| sequence alignment | | D | | - | not relevant | | | | | | | |
| secondary structure analysis of amplicon | | D | | OK | see references for all bacteria and plc gene | | | | | | | |
| location of each primer by exon or intron (if applicable) | | E | | - | not relevant | | | | | | | |
| what splice variants are targeted? | | E | | - | not relevant | | | | | | | |
| **qPCR oligonucleotides** | |  | |  |  | | | | | | | |
| primer sequences | | E | | OK | see all bacteria and plc gene | | | | | | | |
| RTPrimerdb identification number | | D | | OK | see references for all bacteria and plc gene | | | | | | | |
| probe sequences | | D | | - | not relevant, as no probes were used | | | | | | | |
| location and identity of any modifications | | E | | OK | no modifications | | | | | | | |
| manufacturer of oligonucleotides | | D | | OK | Microsynth (Balgach Switzerland) | | | | | | | |
| purification method | | D | | OK | desalted | | | | | | | |
| **qPCR protocol** | |  | |  |  | | | | | | | |
| complete reaction conditions | | E | | OK | See main manuscript | | | | | | | |
| reaction volume and amount of cDNA/DNA | | E | | OK | reaction volume = 20 µl, amount of DNA = 1 µl, hypothetically 1 – 5 ng/µl DNA | | | | | | | |
| primer, (probe), Mg++ and dNTP concentrations | | E | | OK | see manual Brilliant III Ultra-Fast SYBR Green qPCR Master Mix (Agilent, Vienna, Austria), primer = 2.5µM initial concentration | | | | | | | |
| polymerase identity and concentration | | E | | OK | see manual Brilliant III Ultra-Fast SYBR Green qPCR Master Mix (Agilent, Vienna, Austria) | | | | | | | |
| buffer/kit identity and manufacturer | | E | | OK | Brilliant III Ultra-Fast SYBR Green qPCR Master Mix (Agilent, Vienna, Austria) | | | | | | | |
| exact chemical constitution of the buffer | | D | | OK | see manual Brilliant III Ultra-Fast SYBR Green qPCR Master Mix (Agilent, Vienna, Austria) | | | | | | | |
| additives (SYBR green I, DMSO, etc.) | | E | | OK | SYBR Green is included in the supermix; no further additives | | | | | | | |
| manufacturer of plates/tubes and catalog number | | D | | OK | MicroAmp optical tube (0.2 µl; Applied Biosystems by life technologies) | | | | | | | |
| complete thermocycling parameters | | E | | OK | 95°C for 3 min and 40 cycles of 95°C for 5 s followed by 20 s at 61°C, melting curve 70°C to 90°C for all bacteria  see references for *plc* gene | | | | | | | |
| reaction setup (manual/robotic) | | D | | OK | manual | | | | | | | |
| manufacturer of qPCR instrument | | E | | OK | Stratagene Mx3000P real-time PCR System (Agilent Technologies, Santa Clara, USA) | | | | | | | |
| **qPCR validation** | |  | |  |  | | | | | | | |
| evidence of optimisation (from gradients) | | D | | - | published assays were used | | | | | | | |
| specificity (gel, sequence, melt, or digest) | | E | | OK | melting curve | | | | | | | |
| for SYBR green, Cq of the NTC | | E | | OK | no amplification | | | | | | | |
| standard curves with slope and y-intercept | | E | | OK | All bacteria assay:-3.359*LOG(X) + 32.27, *plc* assay: -3.350*LOG(X) + 37.52 | | | | | | | |
| PCR efficiency calculated from slope | | E | | OK | All bacteria assay: 98.5%, *plc* assay: 98.8%. | | | | | | | |
| confidence interval for PCR efficiency or standard error | | D | | - |  | | | | | | | |
| R2 of standard curve | | E | | OK | All bacteria assay: 0.999, *plc* assay: 0.998 | | | | | | | |
| linear dynamic range | | E | | OK | All bacteria assay: 4.49E+01 to 4.49E+07, *plc* assay: 1.39E+03 to 1.39E+06 | | | | | | | |
| Cq variation at lower limit | | E | | OK | 0.22 for all-bacteria assay and 0.46 for *plc* assay. | | | | | | | |
| confidence intervals throughout range | | D | | - |  | | | | | | | |
| evidence for limit of detection | | E | | OK | 10 gene copy numbers for all-bacteria assay and 10^3 bacterial cells/g feces for the *plc* assay. | | | | | | | |
| if multiplex, efficiency and LOD of each assay. | | E | | - | not relevant, no multiplexing | | | | | | | |
| **data analysis** | |  | |  |  | | | | | | | |
| qPCR analysis program (source, version) | | E | | OK | Stratagene Mx3000P real-time PCR System (Agilent Technologies, Santa Clara, USA) | | | | | | | |
| Cq method determination | | E | | OK | Stratagene Mx3000P real-time PCR System settings (baseline subtracted curve fit, single threshold, automatically calculated). Threshold manually curated for maximum efficiency within linear range for each plate | | | | | | | |
| outlier identification and disposition | | E | | - | No outliers | | | | | | | |
| results of NTCs | | E | | OK | no amplificate | | | | | | | |
| justification of number and choice of reference genes | | E | | OK | see references for all bacteria and plc gene | | | | | | | |
| description of normalisation method | | E | | - | not relevant | | | | | | | |
| number and concordance of biological replicates | | D | | OK | three replicate vessels per time point | | | | | | | |
| number and stage (RT or qPCR) of technical replicates | | E | | OK | 2 technical replicates for all samples and for the standard | | | | | | | |
| repeatability (intra-assay variation) | | E | | OK | repeatable | | | | | | | |
| reproducibility (inter-assay variation, %CV) | | D | | OK | not determined (strongly recommended for clinical/diagnostic applications, but not other assays) | | | | | | | |
| power analysis | | D | | - | Not done | | | | | | | |
| statistical methods for result significance | | E | | OK | A linear regression model was applied. Data were assessed visually with regards to normal distribution of residuals (histograms and quantile plots). A contrast coefficient among the six different time points and between the infected and non-infected group were calculated (Tukey). | | | | | | | |
| software (source, version) | | E | | OK | R Team. A language and environment for statistical computing. Foundation for Statistical Computing, Vienna, version 3.1.3. 2015 | | | | | | | |
| Cq or raw data submission using RDML | | D | | - | Not done | | | | | | | |
| E = essential, D = recommended, ^1^ Annealing temperature in °C. | | | | | | | | | | | | |
