# Supplementary material for: The application of rumen simulation technique (RUSITEC) for studying dynamics of the bacterial community and metabolome in rumen fluid and the effects of a challenge with Clostridium perfringens
Source: PLoS One. 2018 Feb 7;13(2):e0192256. doi: 10.1371/journal.pone.0192256 (PMC5802913; doi:10.1371/journal.pone.0192256)
Supplement: S3 Table — OTU richness and diversity between non-infected (A-C) and infected vessels (D-F). (DOCX) [file pone.0192256.s004.docx]

S3 Table. OTU richness and diversity between non-infected (A-C) and infected vessels (D-F).

|  |  | Fermenter | |  |
| --- | --- | --- | --- | --- |
|  |  | A-C | D-F | *P*-value |
| OTU richness | Sampling day 10 | 1523 ± 75 | 1594 ± 191 | NS |
|  | Sampling day 12 | 1486 ± 46 | 1473 ± 26 | NS |
|  | Sampling day 15 | 1390 ± 103 | 1379 ± 54 | NS |
| Chao 1 | Sampling day 10 | 2543 ± 110 | 2646 ± 241 | NS |
|  | Sampling day 12 | 2362 ± 77 | 2423 ± 3 | NS |
|  | Sampling day 15 | 2241 ± 99 | 2243 ± 64 | NS |
| Shannon | Sampling day 10 | 8.03 ± 0.29 | 8.10 ± 0.41 | NS |
|  | Sampling day 12 | 8.17 ± 0.14 | 7.98 ± 0.21 | NS |
|  | Sampling day 15 | 7.77 ± 0.39 | 7.68 ± 0.21 | NS |
| NS = not statistically significant. | | | | |
